# Exploring sleep in farmed ungulates: A scoping review protocol

**DOI:** 10.1371/journal.pone.0343334

**Published:** 2026-02-23

**Authors:** Gustavo Dias, Emma Ternman, Kathryn Proudfoot

**Affiliations:** 1 Department of Health Management, Atlantic Veterinary College, University of Prince Edward Island, Charlottetown, Canada; 2 Department of Production and Welfare, Faculty of Biosciences and Aquaculture, Nord University, Steinkjer, Norway; Kerman University of Medical Sciences, IRAN, ISLAMIC REPUBLIC OF

## Abstract

Sleep is essential to the life of all animals and has been broadly studied in humans and laboratory animals, although less explored in other species. Sleep may influence various aspects of an animal’s welfare, including their health, behaviour and mental states. Some researchers have begun to investigate sleep in farmed ungulates, including the role that sleep may play in animal welfare. This protocol aims to outline the methods of a scoping review aimed at describing sleep studies in farmed ungulates. The proposed scoping review aims to 1) map existing studies on sleep in farmed ungulates, 2) explore the animal welfare implications of this sleep research, and 3) identify knowledge gaps in need of future research. The PRISMA-P guidelines extension for scoping reviews will be followed, and the data will be made publicly available at the time of publication. A search will be performed in three databases: CAB Abstracts, PubMed, and Scopus. Articles will be screened using Covidence software and included if they measured sleep in species of farmed ungulates. The results will be reported using a descriptive synthesis of published sleep studies in farmed ungulates, including a timeline of when studies were published, the species and other characteristics of animals included, methods used to estimate sleep, and how sleep related to animal welfare outcomes.

## Introduction

Sleep is essential to the life of all animals with a nervous system [1]. Total sleep deprivation leads to a behavioral and physiological syndrome resulting in death within 2–3 weeks in rat models [2]. The relationship between sleep, depression, emotions, cognition, health, and physiological status has been an active area of research in humans and laboratory animals for decades [3,4]. There is a growing interest from animal welfare scientists to further explore these relationships in domestic animals, including farmed ungulates [5,6]. As prey species, studying sleep in farmed ungulates may provide insight into evolutionary adaptations that different species have made to maintain adequate sleep, contributing to important comparative research [7].

Sleep may also help us understand how animals cope with their environment, playing a role in the quality of their lives. The welfare of farmed animals is an important area of concern amongst the public [8]. Thus, research focused on identifying metrics that may contribute to improving the welfare of animals is increasingly being prioritized. A common conceptualization of animal welfare is through three concepts described by Fraser (1997), including the animals’ subjective emotions and moods (“affective states”), their health and normal function of biological systems (“biological functioning”), and their ability to express their normal behaviors in their given environment (“natural living”) [9]. Despite growing interest in sleep from animal welfare researchers, there has been no comprehensive synthesis of how sleep has been studied in farmed ungulates and how it relates to animal welfare outcomes.

Evidence from non-human animals indicates a bidirectional relationship between sleep and different aspects of animal welfare. For example, on the one hand, sleep loss can increase disease susceptibility and negative affective states in animals [3,4,10]. On the other hand, disease and negative affective states, such as pain and anxiety, can also alter sleep patterns [3,11,12]. Thus, the relationship between sleep and animal welfare is likely complex and intertwined.

As a growing and complex area of study, a scoping review of sleep research in farmed ungulates is needed to determine the extent to which sleep has been studied and identify areas for future work. The specific objectives of the proposed scoping review will be to: 1) map existing studies on sleep in farmed ungulates (including the period of publication, animal characteristics, and methods used to assess sleep in studies of farmed ungulates), 2) explore the animal welfare implications of this sleep research, and 3) identify knowledge gaps in need of future research. This scoping review protocol aims to define the scope, outline the inclusion/exclusion criteria, establish methodological rigor, and reduce the risk of bias of the proposed scoping review.

## Materials and methods

This scoping review protocol has been developed based on the PRISMA-P guidelines (S1 Appendix). Our objectives align with a scoping review approach as the topic is broad and exploratory. We aim to understand the scope and nature of this research topic, map the existing literature, and identify knowledge gaps, rather than answering a specific research question. The scoping review will follow the PRISMA-ScR guidelines for reporting, including a flow diagram for summarizing the screening and selection process [13].

### Selection criteria

Table 1 outlines the criteria that will be used to determine whether articles would be included or excluded from the scoping review. We opted to only include articles in English due to a lack of translation resources.

**Table 1:** Inclusion and exclusion criteria for accepting or rejecting studies for a scoping review on sleep research in farmed ungulates.

Criterion	Inclusion Criteria	Exclusion Criteria
Population	Studies including commonly domesticated farmed ungulate species (bovine, swine, equine, caprine, and ovine), including those housed in a commercial or research facility	Studies including wild or semi-domesticated ungulates, or animals as models for humans
Context	Studies focusing on sleep or sleep-like behaviour, either as an outcome measurement or an intervention	Studies focusing on lying, resting, drowsing, or another similar concept similar to, but distinct from, sleep
Sources	Peer-reviewed primary articles or meta-analyses published in English after 1950	Grey literature (e.g., abstracts or theses) or secondary sources (e.g., literature reviews)

### Data acquisition and selection

We conducted a pilot study for the scoping review in October 2024. The search strategy was developed with the assistance of a librarian from the University of Prince Edward Island, who has expertise in developing scoping reviews and comprehensive research. In the pilot search, studies were sought in full text across three databases: CAB Abstracts via EBSCOhost, PubMed, and Scopus. Search terms are included in Table 2.

**Table 2:** General search terms of a pilot search for a scoping review on sleep in farmed ungulates to be further adapted to each specific database.

Operator	Concept Search String
Population	Cattle or cow or calf or calves or bull or heifer or steer or pig or swine or sow or hog or boar or piglet or sheep or ewe or ram or lamb or wether or goat or nanny or doe or billy or billies or buck or kid or horse or mare or stallion or gelding or foal or filly or colt or donkey or jenny or jack or jannet or molly or john or pony
Context	Sleep or sleep-like behavior or sleep-like behaviour

**Fig 1:**
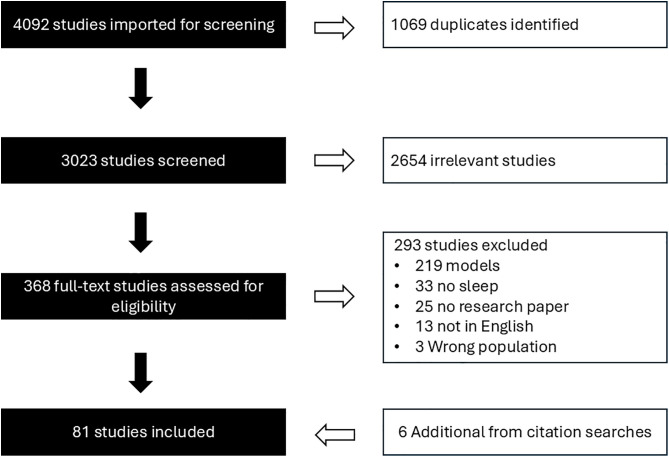
Search and screening process of important studies for a scoping review on sleep in farmed ungulates.

After the initial search, 4,092 articles were imported into Covidence software (Covidence, Melbourne, Australia), where 1,069 duplicate articles were removed (Fig 1). For the pilot search, one primary reviewer (GD) screened the titles, abstract, and full-text of the articles using the inclusion and exclusion criteria. Two co-authors (KP and ET) were consulted to ensure consistency in the application of inclusion and exclusion criteria. In the pilot search, the first screening process removed 2,654 articles based on title and abstract, and the second removed 293 papers after full-text review, resulting in 75 articles. For the scoping review, we will have a secondary reviewer screen a random sample of 20% of the articles to minimize selection bias. Prior to screening, a calibration exercise will be conducted by selecting a sample of 5% of the papers for independent screening by each reviewer. If a 90% or better agreement is not achieved, the reviewers will discuss their points of disagreement, review and revise the inclusion criteria [14].

From the pilot search, using 25% of the 75 articles (n = 19), we performed a hand search of the literature to find any missing articles. To do this, we used Google Scholar to search the references as well as any articles that cited of these 19 articles. Articles used for the hand search were pseudo-randomly selected, ensuring all species were represented in the selected studies. Additionally, we searched for articles in review papers of sleep in farmed ungulates. During the hand search, six articles meeting the review inclusion criteria were added to the preliminary list, resulting in a total of 81 studies included in the pilot (S1 Table). The same search strategy will be used for the scoping review. A new search will be conducted to identify articles published since the pilot search.

### Data extraction and coding

The articles included in the pilot search (n = 81) were classified into two categories by one coder (GD): studies that observed sleep in farmed ungulates without any interventions (observational studies, n = 42) and studies that imposed an intervention that influenced sleep outcomes or where sleep deprivation was the intervention (experimental studies, n = 39). All articles were coded in Microsoft Excel by their title, author(s), year of publication, journal, country, animal type (bovine, swine, equine, caprine, and ovine), animal age (adult, adolescent, infant, fetus, or mixed), animal sex (male, female, or both), breed, number of animals, and method used to estimate sleep (polysomnography, sleep-like behavior, accelerometer, or combination of methods).

Observational studies were further categorized based on their study objectives, including whether they focused on describing sleep, validating sleep methods, assessing physiological changes associated with sleep, or determining environmental changes associated with sleep. Experimental studies were classified according to the intervention using an animal welfare framework (affective states, biological functioning, and natural living), with subcategories under biological functioning (e.g., nutrition, metabolism, stress, disease, and sleep deprivation), natural living (e.g., housing, social, and feeding method), and affective states (e.g., pain).

The same categories and coding will be used for the scoping review. In addition, we will have the secondary reviewer code 20% of the data to ensure consistency using intercoder reliability and a calibration exercise. The calibration exercise for coding will consist of all authors independently extracting data from a random selection of 5 papers and discussing any discrepancies [14]. If needed (i.e., less than 90% agreement), further refinement of the categories will be made.

### Analysis and presentation of results

Two types of descriptive analysis will be carried out in the scoping review. First, we will characterize the studies by publication period, methods used to estimate sleep, and animal characteristics. These data will be presented as graphs and tables, such as a bar graph showing a timeline of studies published from 1950 until now, and a table showing the methods used to assess sleep across different species. Second, we will classify observational or experimental studies based on the researchers’ primary objectives in studying sleep, including their relationship to animal welfare. For experimental studies, this will include a quantitative summary of research that aligns with biological functioning, affective states, and natural living. Additionally, a narrative synthesis will be structured by these different aspects of animal welfare to highlight patterns within and across species and to identify knowledge gaps.

## Discussion

This protocol describes a proposed scoping review with the aims of mapping existing studies on sleep in farmed ungulates, exploring the animal welfare implications of this sleep research, and identifying knowledge gaps in need of future research. To map this literature, we will describe the period of publication, animal characteristics, and methods used to assess sleep in studies of farmed ungulates. The period of publication will allow us to determine trends over time in sleep research and determine whether this is a growing area of scholarly inquiry. An assessment of animal characteristics will help identify any species biases in the work, as well as determine what animal age and sex has been included in current sleep research. As life stage and sex influence sleep in other species [15,16], these factors may be useful for reducing bias in future work assessing sleep in farmed ungulates.

The proposed scoping review will also include a description of methods used to estimate sleep in farmed ungulates. Several methods have been used to assess sleep in animals, including polysomnography [17], sleep-like behaviour [18], accelerometers [19,20], and a combination of physiological indicators such as heart rate and muscle activity [21]. However, not all methods used to assess sleep have been validated. Polysomnography (PSG) is the gold standard method for estimating sleep [17], yet may not be used to researchers because it is labor-intensive and typically requires animals to be housed in restrictive environments to minimize data loss, potentially affecting their natural sleep patterns. In contrast, sleep-like behavior may be easier to measure, but is a poor predictor of sleep in some animals, such as adult cattle [18]. Exploring the methods used to estimate sleep will allow us to assess the consistency of methodology across studies and species, as well as provide a better understanding of which methods have been validated to accurately assess sleep in different species.

To our knowledge, this will be the first scoping review to assess how researchers have studied sleep in animals within a framework of animal welfare. The framework of animal welfare described by Fraser et al. (1997) is relevant for our proposed scoping review, as sleep: 1) supports biological functioning through its role in immunity and disease resistance [4], 2) influences, and can be influenced by, affective states [3], and 3) relates to naturalness, since environmental conditions and the ability to perform normal behaviors shape sleep quantity and quality [22]. Classifying studies using this framework offers insights into future studies at the intersection of animal welfare and sleep and could help guide decisions about housing and management for farmed ungulates to improve their welfare.

We included a pilot study (n = 81) in our protocol as it has been shown to be useful in systematic and scoping review protocols. The primary benefits of a pilot search include helping to test and refine the search strategy, verifying the eligibility criteria, ensuring the feasibility of a review by assessing the volume of literature, and ensuring that the methods are systematically developed and rigorously tested. In this pilot search, we opted to test the coding and classification of studies for observational and experimental studies, and to determine the need for including subcategories. Additionally, the pilot study has helped determine the approach to analyze and summarize the data and will enable us to improve the coding and analysis for the scoping review.

### Limitations

The scoping review will have several limitations. Firstly, due to the restriction of time and resources, we will limit the included databases to three, with the risk of missing papers not indexed in those databases or during the hand-search process. Secondly, the scoping review will only include peer-reviewed research articles or meta-analyses in English and may, thus, lack literature published in other languages or the grey literature. Thirdly, as this is an exploratory review, we will not include or exclude papers based on the methodology used to estimate sleep. Articles using unvalidated methodologies to assess sleep will be included, which means some associations between sleep and outcomes or interventions might not be accurate and will require further investigation. Additionally, assessing sleep patterns through the range of variables presented in observational studies using PSG does not guarantee accurate results, but it is the most precise method due to the lack of research in the area. Lastly, classifying experimental studies using an animal welfare framework might restrict our abilities to describe the authors’ true intentions for their work, biasing the classifications of the papers according to our background.

## Supporting information

S1 Appendix PRISMA-P checklist extension for scoping reviews. (DOCX)

S1 Table List of literature included in the pilot study. (XLSX)
